# *Dark local knowledge*: the yet-to-be scientifically discovered and locally acknowledged aspects of local knowledge systems

**DOI:** 10.1186/s13002-024-00692-x

**Published:** 2024-05-13

**Authors:** Renata Sõukand

**Affiliations:** Department of Environmental Sciences, Informatics and Statistics, Ca’ Foscari University of Venice, Via Torino 155, Venice, Italy

**Keywords:** Dark local knowledge, Traditional practice, Traditional ecological knowledge, Local knowledge, Situated knowledge, Local knowledge systems, Unknown unknown

## Abstract

This essay brings forward the idea that there is more than meets the eye in local knowledge systems than what science can show us now. To comprehend this, we need to make a conceptual jump and look for the “dark matter” (the notion borrowed from astronomy that refers to a hypothetical form of matter that does not interact with light or electromagnetic fields) that can potentially sustain local knowledge. Considering that it is a complex of knowledge, practices, and beliefs contained in TEK, knowledge in LEK does not correspond to the notion of *knowledge* in science. Therefore, in order to map LEK–science interactions, we will refer to the concept of peoples’ knowledge of LEK as *acknowledgement* and the scientific recognition and awareness of information, facts, and principles as *knowledge*. Applying this to a Johari Window, we can observe four categories of LEK in a *known–unknown/acknowledged–unacknowledged* matrix. We can refer to *unknown and unacknowledged* as *dark local knowledge*. Indeed, local knowledge systems contain many aspects that modern science cannot yet explain, as a major part of its components are not even considered in scholarly research. *Dark local knowledge* can potentially provide us with the invaluable touch of experience of countless generations, opening different ways of seeing reality.

The question brought forward by the debate: “Is ethnobiology romanticising traditional practices, posing an urgent need for more experimental studies evaluating local knowledge systems?” has been circulating in scientific discourse for a while in various forms, and Jonathan Coope [7] suggests that the warnings regarding the romanticisation of indigenous cultures, “however well-intentioned, run the risk of unconsciously perpetuating cultural imperialism”. Indeed, in science, the label of romanticisation signals an unscientific approach, and, at the same time, it negatively affects the communities whose practices are studied [8] due to the long history of colonisation of indigenous ecological knowledge [12]. Today, numerous studies prove that local knowledge reveals aspects of reality that science cannot yet explain [34, 44] and I want to take a step forward from there. Local knowledge is a system with all its complex components [4, 41], and to call the strive to study this system from different points of view a romanticisation is not only insulting to the knowledge holders but also dangerous for the credibility of science.

My arguments against the above-mentioned claim are based on my professional, educational, and personal experiences. If I have studied in depth an aspect I address in this debate, I take the liberty to refer to my own work; when covering some other aspects, where I am not aware of the literature, I reflect on my personal (often intuitive) perception. I am a multiethnic and multilingual ethnobotanist with a scientific background in eco-semiotics, environmental science, and pharmacy, who lived traditional practices as a child and experienced the homogenisation of LEK imposed by Soviet occupation. I currently teach international students about the issues of global change and biocultural diversity, directing future environmental scientists and environmental humanities specialists towards a multi-perspective interpretation of reality. In the last ten years, I have spent ca. 30 months in the field in different countries throughout Europe and beyond, experiencing a diversity of practices. I also have felt “something” unexplainable and indescribable, which is reflected in the eyes of people with similar experiences but is not expressed in words. I cannot explain how it “works”, but for me it is a fairly common situation for the interviewee, at the end of the interview, to look deeply into my eyes and say something like “you already know much of what I was telling you, I can understand it from the way you ask questions”. It seems that in ethnobiological research, the depth and breadth of the knowledge that we, as scientists, are able to record depends on the depth of knowledge we already have and the details we choose to take into account or ignore (but only in the event that we can notice them firsthand). In modern scientific writing, there is little space for details, especially in “serious” journals, but the devil is exactly in the details.

Before proceeding to the heart of the question, I want to clarify the terminology. As the phrase “traditional practice” is incorporated in many different terms circulating in the ethnobotanical literature (such as Indigenous/Traditional/Local/Situated knowledge), I will hereafter use the term Local Ecological Knowledge (LEK) to cover all of them. This is because every “piece of knowledge” in the modern world, even in the most remote areas, is influenced by the outside world, either directly or indirectly. Yet, after being applied *in loco*, even introduced knowledge, once established, becomes local and eventually traditional practice for that specific place in a matter of time. To avoid entering into a long discussion on when introduced knowledge becomes traditional, I stress that it is precisely the combination of the locality and the practice that makes the knowledge *local*, and LEK, as a concept, a synthesis of these dynamics from my point of view.

The author of the call for debate supported the statement with specific “negative” descriptive adjectives, claiming local knowledge to often be “fuzzy, hieratic, heterogeneous, contradictory”. I build my response by addressing one by one all of these descriptive adjectives attributed to local knowledge in order to demonstrate why we should study LEK following its own logic.

Fuzziness means being indistinct and without a sharp outline. Obviously, a fuzzy system is difficult to study. Yet, it is the fuzziness of LEK that makes it evolving. Indeed, if something is fuzzy, everyone can interpret it in their own way. Through this, it encodes the possibility for change as well as space for experimenting. From physics, we know that no living system can remain unchanged for a long time; it must change. Therefore, the fuzziness of LEK signals a healthy living system. The unclear borders of LEK provide the potential for continuing experimentation and practice, which can lead to potentially sustainable developments. Whether this potential of sustainable development is realised is another question; it depends on many circumstances that we, as ethnobiologists, specifically study. Given that LEK is formed from long-term experimentation and practices related to the environment in which the community lives, this potential is crucial. If experimentation and practice continue, the community may or may not be able to adapt to changes, depending on a series of factors which are independent of them (like climate or ecosystem change, colonisation, war, economic crisis, etc.). If the borders of LEK were well defined and crystalised, this knowledge would just represent a museum exhibit, not real life. Theoretically, it is possible to re-introduce a documented and “stabilised” practice, but as not all components of practices can be captured in words [6] or even on video, many aspects of LEK will need to be re-discovered through practice.

Hieratic denotes something ritualistic, which is difficult to interpret. The hieratic character attributed to LEK is due to the perceived adherence to age-old practices (although eventually adapting to continuous changes) of some elements of LEK (and probably the assumption that currently life has to change rapidly, and humanity can only advance with innovation). For example, throughout history, cultures that have had problems with food security have developed codes and mechanisms for maintaining the cultural memory of how to obtain nutrition under conditions of food shortage. Such codes can be perceived as hieratical, but every code had a function historically, even if we cannot understand it now. The code can be hidden in (seasonal) rituals, for example, although making acorn bread is mainly a past memory, it is still occasionally prepared for festivities in Afghanistan and Kurdistan [46], and this helps to keep the knowledge and practice in circulation. As long as there is still a critical mass of people and resources able to provide for basic needs, the community should be able to survive (and eventually flourish), even after a large disaster. Now, at a time when we are abandoning within a single lifetime the knowledge and practices developed over hundreds of generations, hieratic elements are greatly needed as rituals can help to carry on knowledge, even in times when it seems plainly abundant [35].

Heterogeneity refers to the consistency of diverse or dissimilar elements. While perceived as negative from the point of view of the repeatability of scientific experiments, heterogeneity is a clear sign of diversity (which we, as ethnobiologists, should strive to protect and nourish) and this is what makes us free and conscious humans. It should not be seen as an obstacle as far as LEK is concerned, as heterogeneity shows that even if the sum of knowledge in society is decreasing, it is still carried forward by some individuals who are practicing the “old way” (learning from family and neighbours) or experimenting with new skills learned in modern society from books and (social) media. We can look at it through the prism of knowledge circulation outlined in Prakofjewa et al. (under review): sustainable knowledge is in motion, circulating within society and changing in different ways.

Homogenous knowledge, in contrast, can exist only in a closed society, which does not have external contacts due to physical (e.g. mountains, deserts, deep forest), informational (nonliterate, no access to the Internet), or political (closed borders, strict regulations, propaganda) barriers restricting the freedom of movement of people and knowledge. While classical ethnobotany often considers cases of physical isolation a plus for bioprospecting, it does not favour the communities in question. Moreover, political isolation by the system which itself contains some artificially promoted elements overlapping or potentially substituting LEK destroys the diversity which existed before. In the DiGe project (www.unive.it/dige), we observed the rapid homogenisation of ethnomedicine (in addition to its erosion) influenced by the biomedical system of the Soviet Union, which also included plants [36]. In this case, homogenisation is a means of controlling the population, and it works on the LEK level as well. If we consider that LEK also takes care of food security and the self-subsistence of a population, it is indeed one of the first targets of a dictatorship, as removing LEK makes communities vulnerable and dependent on centralised supplies and the regime.

Contradictory means mutually opposed or inconsistent. Indeed, it would be much easier to have it all clear and univocal. What a joy it would be to build models with such data! Reality, however, never corresponds to the model. And we know from pharmacology and pharmacognostic experience that a medicine can cure different ailments depending on the dosage or even become a poison in certain conditions and/or concentrations. Moreover, there are many aspects influencing the final effect of a utilised plant in addition to the presence of specific chemicals and molecules. We can list some technical ones, such as conditions of growth, time of collection, methods and extent of processing, and modes of administration; however, not less important is the state of mind of the person using them. While the effect of a placebo is clearly taken into consideration when it comes to biomedical studies, it is rarely accounted for in evaluating contradictory reports, regardless of the canonical work of Daniel Moerman [22]. The growth of personal medicine [32] clearly signals that every single organism is different, and so is the effect a plant has on it. Therefore, there is nothing strange in the contradictory nature of LEK. Indeed, the contradictory character of LEK is written into the constantly changing system by definition, as not all of its components change with the same speed.

Moreover, some perceived contradictions in LEK may also be partially caused by the confusion resulting from poorly prepared scientists in the field (e.g. superficial interviews, or over- and under-differentiation of plants), misidentification [18], misinterpretation of historical sources [37], etc. In the current situation of multisource LEK, it can also be external sources that intentionally mislead [30] or simply provide incomplete information [39]. Eventually, in a living system, “ineffective” uses will be put aside and, in the long term, forgotten, while those with positive feedback (effective either chemically or psychologically) will be continued. While compiling a database of historical ethnomedicine based on Estonian folklore [40], I could clearly observe among the ca. 20,000 records collected over 100 years, the repeating patterns of new plants entering oral traditions and disappearing after a few decades. However, the important part is that the practice continues [35]. When documenting a current situation containing a “glitch” (system error), it is essential that scientists learn to recognise it effectively in order not to pass on erroneous information, as has been done throughout history [14, 42].

The author of the call for the debate suggested that we need more experimental studies that can verify which elements of LEK are suitable or useful for society. Those are certainly needed and reassuring, but a recent study has shown that the interpretation of scientific datasets is nevertheless subjective [25]. Moreover, are we, with our current research abilities, really able to access every single piece of knowledge we suspect is effective or ineffective? In the last thirty years, over 100,000 studies on medicinal plants have been published, focusing mainly on the search for new medicines or active compounds and leaving all the other aspects far behind [33]. Many elements of current LEK have been the result of long-term experiments conducted over time, and, not without reason, many European institutions (like EMA www.ema.europa.eu) consider evidence of generation-long use sufficient proof of effectivity (although this is not always justified, [38]).

The scope of LEK studies goes far beyond proving a plant’s chemical efficacy in treating specific diseases. Our discipline is growing and is becoming increasingly important in scientific as well as societal discourse. We are not only studying why and how people use plants and other biota. Ethnobiology has developed interdisciplinary instruments (borrowing and combining them from different scientific fields) and has added trans-disciplinary dimensions by involving IP and LC in research. This allows us to tackle burning societal challenges such as the loss of biodiversity [43], climate change [10], plant blindness and naturophobia guided by the extinction of experience [29], mental health issues due to the lack of contact with nature [45], and many more.

As LEK is unequally represented in mainstream scientific discourse, as highlighted by Obura et al. [24] for conservational sciences, we also need to make our field results more visible to put pressure on policymakers to support the sustainability of local communities [2]. To gain the most from our discipline, we need to reformulate the goals of ethnobiology: we should still document and analyse LEK, yet we need to make a conceptual jump. We should not restrict ourselves to scientific hypotheses [31] or qualitative descriptions, even if those form an excellent basis for in-depth analysis [17]. We need to find the common elements and patterns that could help us to foster this relationship and continue to practice the knowledge that supports life (both human and non-human) in every place on earth. We need to encourage those who carry knowledge to pass it on to the next generation, creating a critical mass in every community that carries along resilience in relation to the environment in which the community lives.

## There is more to LEK than meets the eye

Local communities have somehow survived and have taken care of their environment since long before nature conservation and grocery stores based on intensive farming were invented, and therefore, they may know something we still need to learn. Biosemiotician Timo Maran repeatedly stresses in his recent book (2020) that current environmental issues are derived from a semiotic problem, the result of semiotic pollution, or the corrupt way we relate to the ecosystem. Maran refers to Kalevi Kull, outlining the motivation of our actions by “our sign-based distinctions” and stressing that the impoverishment of the ecosystem is the result of self-contained culture [21: 2]. Basing his argument on Juri Lotman’s communication theory and Gregory Bateson’s epistemology of the sacred, he proposes that “normal functioning of culture depends on the dialogue with what lies outside of cultural codes and hierarchies”, indicating the necessity to support interactive practices with “the rest of ecosystem” (Ibid). Maran emphasises the importance of tacit knowledge (an idea framed by Hungarian philosopher Michael Polanyi as a kind of pre-linguistic knowledge acquirable only through participation, living through the process) and its impact on our relationship with nature (Ibid: 27). Indeed, all the knowledge and skills acquired from the environment are of very participatory origin and without an actual hands-on approach the relationship cannot function. Maran refers to Sebeok stressing: “humans are semiotically rooted in nature” (Ibid: 29), yet those roots tend to fade in modern lifestyle.

In ethnobotany, the majority of scientific publications speak about explicit (languaged) knowledge: it is easy to document. Tacit knowledge cannot be easily documented [6], as it is learned only through person-to-person instruction or through personal experience/experimentation. It may not have any names or it may have them in some cultures or languages but not in others. The opportunities to express tacit knowledge through the explicit are limited, as not all experiences can be named. Culture and language are developed in a specific environment and shape the way we perceive ourselves and (in) that or any other environment.

**Fig. 1:**
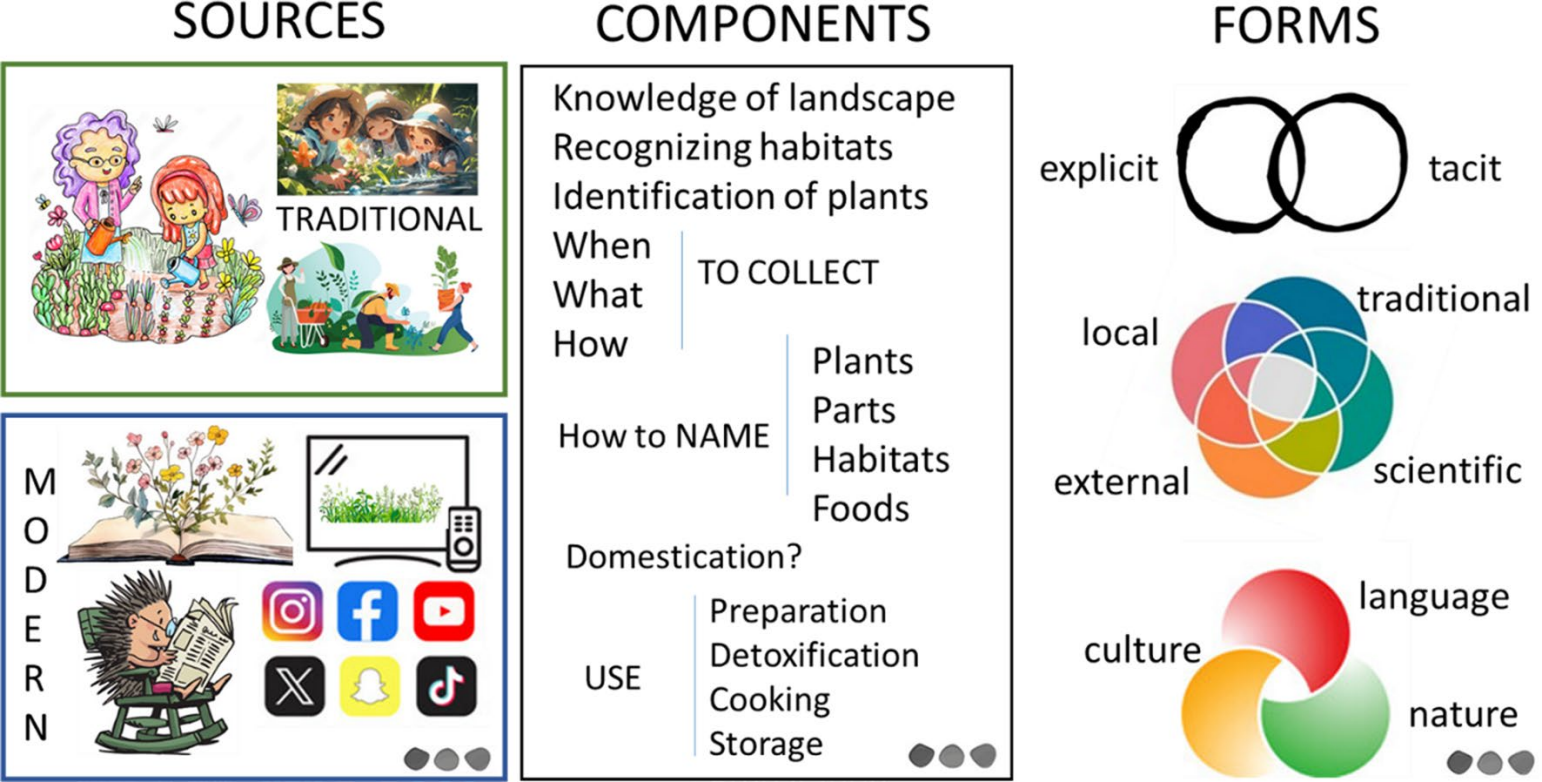
Complexity of local foraging knowledge. Designed with adobe stock

Let’s take foraging as an example. Some aspects of the explicit form of foraging practices (like plant names and the names of the local dishes) are intensively studied and addressed in the majority of wild food plant-related research. Recently, the ethno-organoleptic properties of wild plants have also been covered [28]. At the same time, ethnoecological terminology (like names of habitats or types of landscape) is very rarely addressed [23]. Even in these limited studies, many aspects have been largely neglected (like local names of plant parts, different levels of readiness of plants for consumption, references to poisons, to name only a few). The corpora of LFK components are much more diverse (Fig. 1) and have not yet been completely mapped, even for languaged forms of LFK.

Since February 2002, when then US Secretary of State for Defence Donald Rumsfeld highlighted the “unknown unknowns” for political decisions, the concept has become viral. The existence of the *unknown unknown* has already been acknowledged in experimental biology [15], although in relation to ethnobiology only the level of the *known unknown* has been recognised, introduced by other disciplines [13]. The idea has long been used in business management and derives from the field of cognitive psychology: in 1955, two American psychologists, Joseph Luft and Harrington Ingham, developed the visual tool called the Johari Window [20]

Using the original Johari Window as a matrix, I adapt it to LEK studies in a way that opens a new perspective. However, we need to keep in mind that even if the notion of knowledge is widespread for LEK, we should not forget the initial definition of TEK: as the complex of knowledge, practices, and beliefs contained in TEK [3] does not correspond to the notion of *knowledge* in science. Therefore, in this context, I suggest talking about the *acknowledgement* of LEK elements by people and leaving the *knowledge* as a term when we speak about science in the context of Johari Window. Knowledge is the understanding or awareness of information, facts, or principles (e.g. scientific component), whereas acknowledging is the act of recognising or accepting the existence of specific knowledge by people. In simpler terms, what I hereafter call *known* refers to what science knows, while *acknowledged* refers to the reflection of people on their practices related to the environment. So, LEK–science interactions can be divided into four categories on a *known–unknown/acknowledged–unacknowledged* scale (Table 1).

**Table 1:** Interpretation of LEK–science relations inspired by the Johari Window

	Acknowledged by people	Unacknowledged by people
Known to science	Known and acknowledged	Known but unacknowledged
Not known to science	Unknown but acknowledged	Unknown and unacknowledged

*Known and acknowledged* is LEK which people refer to as to their own (e.g. whose existence is acknowledged) and realise its value, while it makes sense for science as well (e.g. it is scientifically comprehended and/or documented). It refers to scientifically proven (or accepted) elements of LEK that have become common knowledge and seemingly hold no potential interest in science (like long-tested or described medicinal plants or widely used wild food plants). It is kind of boring, but there are many publications that consider long-proven trajectories, which are still needed in order to see the “big picture”.

The *known but unacknowledged* category consists of components of LEK which are studied by scientific discourse but not considered important to people, even if those elements are part of their everyday routine. Sometimes I sense people’s genuine interest in trying to understand why such highly educated guests are interested in what they had for dinner or how they search for a plant while they are instead offering to list for us the medicinal plants they have learned from a TV show or book which they value highly at a specific moment. Those are elements of LEK that we, as scientists, study without people actually giving them value. A major part of hypothesis-driven scientific investigation covers this category. We study what we find interesting for science, even thinking sometimes that we do science for people. Our goal is to prove or deny the existence of the phenomena that we, scientists, know or think exist. We assume that LEK contains some knowledge that may be able to help to solve humanity’s challenges; we just need to understand which and how. The easiest example is related to bioprospecting: by screening for bioactive molecules in exotic medicinal plants with certain characteristics, we can evaluate the level of their efficacy even if people use them for other purposes.

*The unknown but acknowledged* category can also be covered by science, but more on the qualitative side. This is new knowledge for science, which is discovered when science is done with people or as a result of long-term observations and participation in community life. Rarely, the elements belonging to this category can be discovered as a side-product of hypothesis-driven research, but most often they are simply ignored or deemed unimportant (like ad hoc names, blurred elements of knowledge, new book-based uses, and many more).

The *unknown and unacknowledged* is the most interesting category for our discussion. Science has no idea that it exists, as it cannot yet comprehend it from a scientific point of view). People also do not acknowledge its existence (in a way that science could grasp it), although it may be an integral part of their practices and beliefs. Being unknown and unacknowledged, these elements cannot be named, for now. The moment either science or people account for any of it, such an element automatically moves into one of the other three categories.

To start talking about these unknown and unacknowledged elements, we would need to name them first. In ecology, there is a relatively recent concept of *dark diversity*, which encompasses the diversity that cannot be observed (e.g. missed by sampling performed in a specific place) yet affects the observable diversity and helps to understand the composition and dynamics of ecological communities [26]. The idea of dark diversity is derived, although a bit unconnectedly, from the concept of dark matter in astronomy, which corresponds to 90% of all matter in the universe and whose discovery and description caused a scientific revolution [9]. Although dark knowledge has already been defined as something hidden and forbidden [5], we could use the term *dark local knowledge* (DLK) to describe the *unknown and unacknowledged* aspects of LEK. DLK can potentially provide us with the invaluable touch of experience of countless generations, opening for us different ways of seeing reality. *Dark local knowledge*, thanks to the hieratic nature of LEK, may still possess yet unknown aspects supporting the sustainability of human life.

One may question that if DLK is *unknown and unacknowledged*, how can we find it and if we need to do it? Here, we should not forget about the practice and believes contained in the definition of LEK and should rely on the theory of partial overlap defined by David Ludwig [19], suggesting the importance of failures alongside successes in knowledge integration of Indigenous and Western scientific ontologies, as full integration often fails to accept Indigenous standpoints. In his path-breaking book *Culture and Explosion* [16], semiotician Juri Lotman, in the chapter on monolingual systems, describes the communication process as the exchange of information through the points of reference jointly shared by the two communicating entities. Yet, dialogue is possible only as the result of the existence of the non-intersecting parts of the knowledge systems of two communicating entities: the more diverse they are, the more productive the communication is. Without involving non-intersecting parts, the dialogue turns into a monologue, as both entities know what it is all about and when the otherness is exhausted, the exchange is no longer possible, and diversity cannot be celebrated. Exchange and tolerance of being different can produce, instead, higher diversity for both humans and the environment they inhabit; for example, multi-cultural co-existence in Iraqi Kurdistan created high diversity in gathered wild vegetables as there was space for every culture to develop along its own trajectory [27]. A richness which, even in the face of war, has survived and gives hope to people.

Local Knowledge System studies are badly needed to understand and reinforce our relationship with the environment. Because of frightening “end of the world” narratives and losing touch with ourselves and with the environment, humanity faces a “myth gap” [11]: facts and arguments are not enough, we again need stories that tell us who we are, where we live, and where we are going. LEK (and DLK) may provide the tools to help overcome this gap, supporting local communities and offering positive examples of reciprocal coexistence and restoring relationships with and within the environment. Our goal is to amplify and resonate “shared stories that become real, create context, meaning and shared purpose for framing decisions and guiding action” [1].

## Conclusion

The qualities put forward as disadvantages of LEK are in fact its strengths and potential to generate value thanks to DLK. Clearly, even DLK might not help to solve all the problems humanity faces, yet it may help to better address some of them, especially if studies are designed for the benefit of and together with IPLC still able to “read” the environment they live in.

DLK can potentially provide us with the invaluable touch of experience of countless generations, opening for us different ways of seeing reality. *Dark local knowledge*, thanks to the hieratic nature of LEK, may still possess yet unknown aspects supporting the sustainability of human–nature interactions, literally our roots. Our job is to keep the diversity alive, e.g. DLK continues circulating until we are able to learn how to read and translate it. We also need stories that link the knowledge of scientists with LEK, at the same time avoiding overwriting it.

Just as in ecology, the missing dark biodiversity holds up the present biodiversity; just as in astrology, dark matter holds up the visible matter, holds DLK up the LEK as we know it. Luckily, regardless of our discussions and intellectual exercises, as long as there are people who are engaged with the environment, LEK evolves relying on the DLK whose existence we, scientists, are yet to discover.

## Data Availability

Not applicable.

## Abbreviations

DLK: Dark local knowledge
IPLC: Indigenous people and local communities
LEK: Local ecological knowledge
